# Regulation of the Sar1 GTPase Cycle Is Necessary for Large Cargo Secretion from the Endoplasmic Reticulum

**DOI:** 10.3389/fcell.2017.00075

**Published:** 2017-08-23

**Authors:** Kota Saito, Miharu Maeda, Toshiaki Katada

**Affiliations:** Department of Physiological Chemistry, Graduate School of Pharmaceutical Sciences, University of Tokyo Tokyo, Japan

**Keywords:** SAR1, Sec12, COPII, ER, collagen, cTAGE5, Tango1, Sec16

## Abstract

Proteins synthesized within the endoplasmic reticulum (ER) are transported to the Golgi via coat protein complex II (COPII)-coated vesicles. The formation of COPII-coated vesicles is regulated by the GTPase cycle of Sar1. Activated Sar1 is recruited to ER membranes and forms a pre-budding complex with cargoes and the inner-coat complex. The outer-coat complex then stimulates Sar1 inactivation and completes vesicle formation. The mechanisms of forming transport carriers are well-conserved among species; however, in mammalian cells, several cargo molecules such as collagen, and chylomicrons are too large to be accommodated in conventional COPII-coated vesicles. Thus, special cargo-receptor complexes are required for their export from the ER. cTAGE5/TANGO1 complexes and their isoforms have been identified as cargo receptors for these macromolecules. Recent reports suggest that the cTAGE5/TANGO1 complex interacts with the GEF and the GAP of Sar1 and tightly regulates its GTPase cycle to accomplish large cargo secretion.

## Introduction

Eukaryotic cells are characterized by elaborate inner structures called organelles, which allow different functions to be compartmentalized in different regions of the cytoplasm. Proteins and lipids are transferred between these compartments by membrane trafficking. Secretory proteins synthesized within the endoplasmic reticulum (ER) pass through the Golgi apparatus en route to the cell surface (Palade, 1975). Transport from the ER to the Golgi is mediated by coat protein complex II (COPII)-coated vesicles, which typically have diameters of 60–90 nm, and bud from specialized ER domains called ER exit sites or transitional ER (Barlowe et al., 1994). Genetic screening in the budding yeast *Saccharomyces cerevisiae* (*S. cerevisiae*) identified proteins involved in COPII vesicle formation and further biochemical studies revealed the sequence of the events (Novick et al., 1980; Kaiser and Schekman, 1990; Barlowe et al., 1994; Matsuoka et al., 1998).

COPII vesicles comprise small GTPase Sar1, the inner-coat complex (Sec23/Sec24) and the outer-coat complex (Sec13/Sec31). The formation of COPII-coated vesicles initiates with the activation of Sar1 by its guanine-nucleotide exchange factor (GEF), Sec12 (Barlowe and Schekman, 1993; Barlowe et al., 1993). The activated Sar1 recruits Sec23 and Sec24 heterodimers to form the pre-budding complex with cargo proteins (Matsuoka et al., 1998; Bi et al., 2002; Sato and Nakano, 2005). Sec13/Sec31 then binds to Sec23/Sec24, and this interaction enhances the GTPase-activating protein (GAP) activity of Sec23 toward Sar1, and completes the coat assembly (Yoshihisa et al., 1993; Antonny et al., 2001; Bi et al., 2007).

Sec16, another peripheral membrane protein required for COPII vesicle formation *in vivo*, is conserved from yeast to human (Kaiser and Schekman, 1990; Connerly et al., 2005; Watson et al., 2006; Iinuma et al., 2007; Ivan et al., 2008). Sec16 interacts with several COPII proteins (Gimeno et al., 1996; Shaywitz et al., 1997; Bhattacharyya and Glick, 2007; Hughes et al., 2009; Whittle and Schwartz, 2010; Montegna et al., 2012), indicating that it organizes and acts as a scaffold for COPII assembly (Sprangers and Rabouille, 2015). Of note, a recent study using the budding yeast *Pichia pastoris* (*P. pastoris*) proposed that Sec16 acts as a regulator of COPII assembly rather than a scaffold (Bharucha et al., 2013). This model is based on the observation that Sec16 localizes at the ER exit sites via interaction with Sec23. Conversely, Sec23 depletion does not affect the localization of Sec16 at the ER exit sites in *Drosophila* and mammalian cells (Watson et al., 2006; Bhattacharyya and Glick, 2007; Iinuma et al., 2007; Ivan et al., 2008). Thus, Sec16 seems to have diverse properties among species.

## Sar1 GTPase

Sar1 is an Arf family small GTPase initially identified as a multicopy suppressor of a temperature-sensitive *Sec12* mutant in yeast (Nakano and Muramatsu, 1989). Although yeast have a single *Sar1* gene, vertebrates have two (*Sar1A* and *Sar1B*), and the model plant *Arabidopsis thaliana* contains four (*AtSARA1a, AtSARA1b, AtSARA1c, AtSARA1d*). The function of Sar1 in plants is extensively reviewed in recent literature (Yorimitsu et al., 2014).

A role for Sar1 in formation of COPII-coated vesicles is clear, however, the mechanism by which it contributes to cargo concentration, vesicle budding, and scission is still debated. An *in vitro* reconstitution assay revealed that the budding of COPII-coated vesicles requires GTP loading of Sar1 (Barlowe et al., 1994) and that the GTPase activity of Sar1 is necessary for vesicles to detach from the ER (Antonny et al., 2001; Bielli et al., 2005; Bacia et al., 2011). However, several reports have indicated that the GTPase activity is not necessarily required for this process (Adolf et al., 2013). Further studies suggest that the GTPase cycle of Sar1 is not coupled with vesicle budding and continuous cycles induce cargo concentration in nascent COPII vesicles (Futai et al., 2004; Tabata et al., 2009). Biochemical studies using synthetic liposomes have revealed that the N-terminal amphipathic helix of Sar1 penetrates ER membranes and induces membrane curvature upon activation (Lee et al., 2005; Long et al., 2010). Moreover, an *in vitro* tether-pulling rigidity assay with optically trapped microspheres indicated that Sar1 introduction lowered membrane rigidity (Settles et al., 2010). Interestingly, a recent biochemical study suggests that membrane curvature also increases the affinity of Sar1 to membranes and enhances its GTPase activity, indicating the existence of a positive feedback loop of the Sar1 GTPase cycle for vesicle formation (Hanna et al., 2016). Taken together, Sar1 acts as a multi-functional protein required for COPII-vesicle formation.

## Sec12 as a GEF for Sar1 GTPase

Sec12 was first identified by a complementation assay with a temperature-sensitive secretory mutant isolated by genetic screening (Novick et al., 1980; Nakano et al., 1988). Sec12 is a type II transmembrane protein with a WD40 motif and is required for Sar1 membrane localization (D'enfert et al., 1991a,b; Chardin and Callebaut, 2002). Further characterization revealed that Sec12 acts as a GEF for Sar1 and is required for membrane budding from synthetic liposomes (Barlowe and Schekman, 1993; Futai et al., 2004). Recently, a crystal structure revealed that a potassium ion is essential for optimum GEF activity (McMahon et al., 2012). In *S. cerevisiae*, Sec12 was observed by both conventional and super resolution confocal microscopy to be localized throughout the ER (Nishikawa and Nakano, 1993; Okamoto et al., 2012). In contrast, Sec12 in another budding yeast *P. pastoris* localizes to the ER exit sites by direct binding to Sec16 (Soderholm et al., 2004; Montegna et al., 2012). Weissman et al. have shown that the mammalian Sec12 is a GEF for Sar1 localized throughout the ER (Weissman et al., 2001). Recently, it has been reported that Sec12 is concentrated at the ER exit sites by its direct interaction with cTAGE5, a protein required for collagen export from the ER (Saito et al., 2014). Noteworthy, mammalian Sec12 was originally identified as a DNA-binding protein that regulates *prolactin* promoter activity, and was designated Prolactin Regulatory Element Binding protein (Fliss et al., 1999). The relationship between the distinct function of the protein as a transcription regulator and an activator of ER to Golgi transport remains unresolved.

## Sec23, a GAP for Sar1 GTPase, forms inner coat complex with Sec24

Sec23 forms a stable complex with Sec24 and acts as an inner-coat complex for COPII (Hicke et al., 1992). In addition, Sec23 possesses GAP activity toward Sar1 (Yoshihisa et al., 1993), which is drastically enhanced by its interaction with Sec13/Sec31 (Antonny et al., 2001; Bi et al., 2007). On the other hand, Sec24 acts as a cargo adaptor and interacts directly with membrane-spanning cargo proteins or indirectly with cargo receptors, which then interact with secretory cargoes by their luminal regions. Sec24 has multiple binding sites for cargo proteins and the specificity of interaction between cargoes and Sec24 isoforms enables discrimination of cargo proteins entering into the forming pre-budding complex (Miller et al., 2002, 2003; Mossessova et al., 2003).

Sec16 modulates Sar1 GTPase activity either by interacting with Sec24 or by preventing the recruitment of Sec31 to the pre-budding complex (Kung et al., 2012; Yorimitsu and Sato, 2012).

## The Sar1 GTPase cycle is involved in large carrier formation

Several cargoes secreted by mammalian cells such as collagen and chylomicrons are larger than conventional coated vesicles. Emerging evidence suggests that transport of large cargoes through the Golgi apparatus is mediated by cisternal maturation, so that large cargoes do not need to be accommodated by COPI-coated vesicles for anterograde intra-Golgi trafficking (Nakano and Luini, 2010; Glick and Luini, 2011). Conversely, export from the ER has been reported to be a COPII-dependent process (Stephens and Pepperkok, 2002; Fromme and Schekman, 2005). Recent immunofluorescence and electron microscopy analysis revealed that these extra-large cargoes are accommodated by large COPII vesicles (Gorur et al., 2017), yet the mechanisms of large vesicle formation have not been fully elucidated (Stagg et al., 2006). One promising source for these large vesicles has been observed by electron microscopy as a protrusion of the ER adjacent to the ER exit sites (Mironov et al., 2003). Interestingly, when semi-intact cells are incubated with a GTP-restricted form of Sar1 (Sar1 H79G), tubes emanate from the ER (Aridor et al., 2001). Similar tubule formations are also found in artificial liposomes incubated with Sar1 H79G or with Sar1 in the presence of non-hydrolyzable GTP analogs such as GTPγS and GMP-PNP (Antonny et al., 2001; Bielli et al., 2005; Long et al., 2010; Bacia et al., 2011). Moreover, Zanetti et al. have shown by cryo-electron microscopy that giant unilamellar vesicles incubated with non-hydrolyzable Sar1 and COPII components induced tubes covered with Sec23/24 and Sec13/31 (Zanetti et al., 2013). Interestingly, the model predicts that a tubular structure coated with Sec23/24 recruits less Sec13/31 than spherical vesicles, implying that expanded COPII cages may have a reduced Sar1-GTP hydrolysis activity compared with conventional COPII coats. These results are consistent with the observation that Sar1 recruitment to the membrane induces membrane curvature and suggests that regulation of the Sar1 GTPase cycle is important for forming large COPII carriers. Indeed, recent evidence suggests that some of the factors specifically required for large cargo secretion involve the regulation of the Sar1 GTPase cycle (Saito and Katada, 2015).

Of interest, mutations in the Sar1B nucleotide binding regions lead to genetic diseases such as the chylomicron retention disease, and Andersen disease, characterized by defects in transport of large chylomicrons, suggesting that Sar1B is required for large cargo secretion (Jones et al., 2003). A similar phenotype was also observed in a fish model with morpholino-based knockdown of Sar1B (Levic et al., 2015). As Sar1A and Sar1B share 89% sequence identity, the specific Sar1B perturbation phenotype is surprising. One explanation comes from the observation that Sar1B is the predominant isoform expressed in the intestine and liver, where chylomicrons, and very low density lipoproteins are secreted. Moreover, Sar1B expression specifically promotes ApoB-containing lipoporotein secretion, whereas Sar1A expression antagonizes the effect. Depletion of Sar1B, but not Sar1A, specifically reduces ER export of Srebp2 (Fryer et al., 2014). Biochemically, an *in vitro* tether-pulling rigidity assay suggests that the Sar1B-mediated COPII coats are looser than those with Sar1A (Loftus et al., 2012). The mechanisms of Sar1B-specific chylomicron secretion from the ER require further investigation.

## Efficient activation of Sar1 GTPase is required for large cargo transport

**Figure 1:**
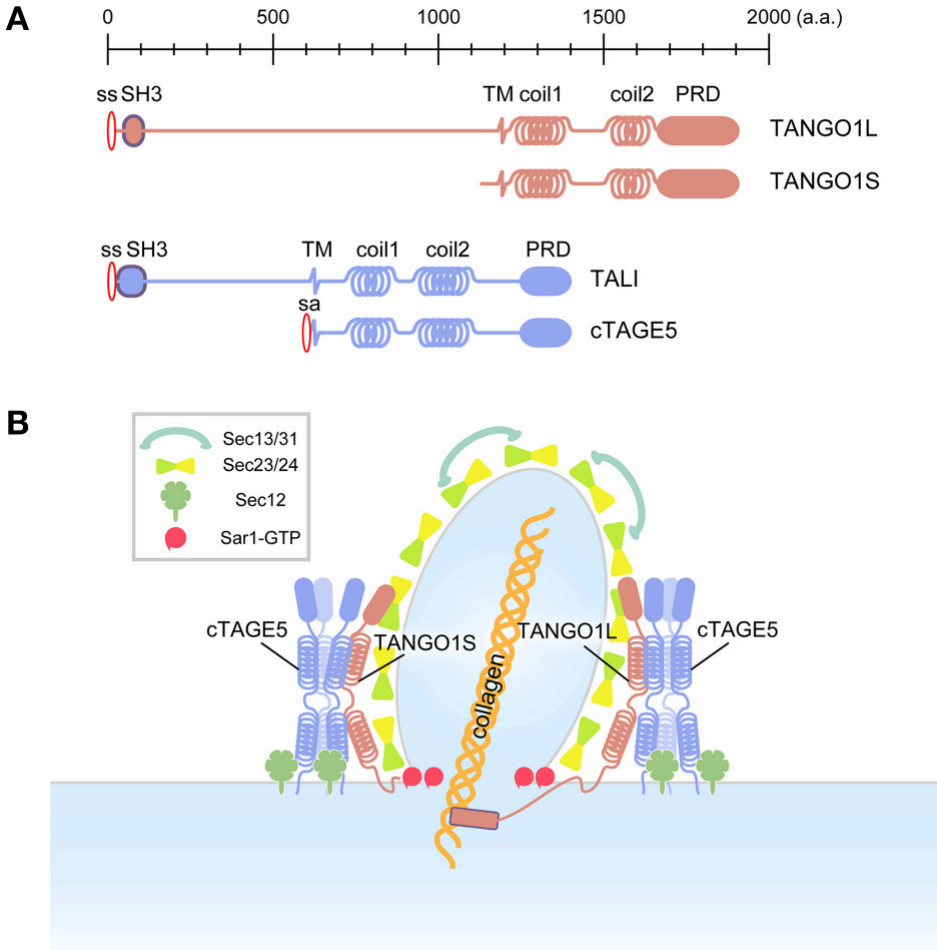
Schematic of conventional and collagen-containing large COPII-vesicle formation. **(A)** Domain organization of the mammalian TANGO1 family. **(B)** In contrast to conventional COPII vesicles, the large COPII structure requires the TANGO1/cTAGE5/Sec12 complex, which efficiently activates Sar1 GTPase in the vicinity of forming large COPII structures.

Genome wide screening in *Drosophila S2* cells led to the identification of factors involved in ER to Golgi transport. These factors were termed Transport ANd Golgi Organization (TANGO) (Bard et al., 2006). Among them, TANGO1 is required for collagen secretion (Saito et al., 2009; Wilson et al., 2011). In mammals, *TANGO1* gives rise to two alternative splicing variants, TANGO1L, and TANGO1S (Wilson et al., 2011) (Figure 1A). Although both proteins are integral membrane proteins localized at ER exit sites, TANGO1S lacks the long luminal sequence present in TANGO1L (Maeda et al., 2016). The luminal SH3 domain of TANGO1L has been shown to interact with collagen either directly or through heat shock protein 47 (HSP47), a chaperone specific for collagen folding (Saito et al., 2009; Ishikawa et al., 2016). The cytoplasmic proline-rich domain (PRD) of TANGO1 (in both variants) directly interacts with Sec23 (Saito et al., 2009; Ma and Goldberg, 2016), and this interaction is supposed to inhibit the recruitment of the Sec13/31 complex, thereby forming large COPII-coated carriers (Malhotra and Erlmann, 2015; Saito and Katada, 2015; Ma and Goldberg, 2016). Thus, TANGO1L acts as a cargo receptor for collagen, as exemplified by TANGO1L-knockout mice exhibiting severe defects in collagen secretion (Wilson et al., 2011). It is noteworthy that not only TANGO1L, but also TANGO1S is required for collagen export from the ER (Maeda et al., 2016) (Figure 1B). We reasoned that the property of TANGO1S to recruit Sec12 to the ER exit sites via cTAGE5 (See below) is critical for collagen secretion despite lacking the collagen recognition domain. In addition, TANGO1 has been reported to interact with Sly1, a Sec1/Munc18 family protein involved in SNARE-mediated fusion that recruits ER-Golgi intermediate compartment (ERGIC) membranes to the ER exit sites for large COPII vesicle formation (Nogueira et al., 2014; Santos et al., 2015).

cTAGE5, a different gene product with the same domain organization as TANGO1S, directly interacts with TANGO1 via the second coiled-coil domains of both proteins (Saito et al., 2011) (Figure 1A). cTAGE5, but not TANGO1, directly binds to Sec12 via a cytoplasmic region just after the membrane spanning domain (Saito et al., 2014; Tanabe et al., 2016). Further biochemical analysis suggests that these proteins form two distinct macromolecular complexes at the ER exit sites: (i) ~900 kDa complex consisting of TANGO1L, a cTAGE5 multimer, and a corresponding amount of Sec12 interacting with cTAGE5; and (ii) ~700 kDa complex consisting of TANGO1S, a cTAGE5 multimer, and a corresponding amount of Sec12 (Maeda et al., 2016) (Figure 1B). Interestingly, Sec12 concentrates at the ER exit sites via interaction with cTAGE5 and this concentration is specifically required for collagen export from the ER (Saito et al., 2014). The interaction with cTAGE5 does not seem to exert any influence on the activity of Sec12 toward Sar1 (Saito et al., 2014). However, further analysis suggests that the Sec12 concentration is required for efficient activation of Sar1 in the vicinity of the ER exit sites (Tanabe et al., 2016). Recently, it has been reported that the interaction between cTAGE5 and Sec12 is also required for the enlargement of ER exit sites during starvation, and that it modulates autophagosome biogenesis (Ge et al., 2017).

Interestingly, cTAGE5 has tissue-specific long isoforms, which contain a luminal region like TANGO1L, and designated TANGO1-like (TALI) (Figure 1A). TALI interacts with TANGO1 as cTAGE5 does, and both TANGO1 and TALI interact with ApoB and are involved in ApoB secretion from the ER, confirming the roles of the TANGO1 family in large cargo secretion (Pitman et al., 2011; Santos et al., 2016). Although not proven experimentally, yet, it is easy to speculate that TALI also interacts with Sec12 and has a role in concentrating activated Sar1 in the proximity of ER exit sites.

In conclusion, mammalian ER exit sites possess macromolecular complexes, which enable efficient Sar1 activation around ER exit sites, and this activity is specifically required for collagen secretion from the ER.

## Regulation of Sar1 GTPase activity is required for large cargo secretion

Human Sec23A missense mutations lead to a rare autosomal recessive syndrome called cranio-lenticulo-sutural dysplasia (CLSD), characterized by facial dysmorphisms, skeletal defects, late-closing fontanels, and cataracts. These clinical characteristics suggest abnormal formation of bones and connective tissues, implying that collagen deposition is perturbed. This is supported by the observation that fibroblasts from these patients accumulate collagen I within the ER (Boyadjiev et al., 2006, 2011). The defects in collagen secretion due to Sec23A perturbation were also confirmed in Sec23A-deficient mice, which show aberrant secretion of collagen, a phenotype similar to that of TANGO1L and a fish mutant carrying a non-sense mutation in *Sec23A* called *crusher*, which displays craniofacial defects (Lang et al., 2006; Zhu et al., 2015). The specific defects in collagen secretion due to Sec23A disruption may be explained by the tissue-specific distribution of the Sec23 isoforms, Sec23A and Sec23B (Fromme et al., 2008). Two point mutations (F382L, M702V) have been found within Sec23A in CLSD patients and both of the mutations have been extensively characterized biochemically. F382L-Sec23A has been shown to impair *in vitro* vesicle budding, probably because it failed to recruit the outer-coat complex, Sec13/31 (Fromme et al., 2007). Conversely, M702V-Sec23A formed vesicles normally in an *in vitro* assay and was capable of interacting with other COPII components to the same extent as wild type Sec23A (Kim et al., 2012). The defects were observed when the GAP activity of these mutants was analyzed in the presence of Sec13/31. When both mutants complexed with Sec24 were incubated with Sar1, they showed GAP activity comparable with that of wild type Sec23A for both Sar1A and Sar1B. Even in the presence of Sec13/31, both mutants showed enhanced GAP activity toward Sar1A as observed with wild type Sec23A; however, when incubated with Sar1B, Sec13/31 failed to stimulate the GAP activity of the Sec23A-F382L mutant. In contrast, incubation with Sec13/31 aberrantly stimulated the GAP activity of Sec23A-M702V toward Sar1B compared with that of wild type Sec23A. These data strongly suggest that appropriate regulation of Sar1 GTPase activity is required for collagen secretion.

Sec31 has also been shown to be involved in large cargo secretion. Recent studies have revealed that two calcium-binding proteins, ALG2 and PEF1, control Cul3-KLHL12-dependent ubiquitylation of Sec31 by calcium signals, and ubiquitylated Sec31 leads to the formation of large COPII carriers (Jin et al., 2012; McGourty et al., 2016). Whether Sec31 ubiquitylation has any effect on the GAP enhancing activity of Sec31 remains to be investigated.

Another protein involved in the regulation of Sar1 GTPase activity is Sedlin, a component of the TRAnsport Protein Particle (TRAPP) complex, also known as TRAPPC2. Sedlin is mutated in a human disease called spondyloepiphyseal dysplasia tarda (SEDT), an X-linked skeletal disorder characterized by short stature and degenerative joints (Gedeon et al., 1999). Sedlin interacts with both TANGO1 and the activated form of Sar1. Depletion of Sedlin causes the accumulation of the activated form of Sar1 at ER exit sites and blocks collagen secretion, implying that Sedlin inactivates Sar1 either directly or indirectly, and this inactivation may be required for large cargo secretion (Venditti et al., 2012).

Taken together, although it has not been fully revealed yet how the Sar1 GTPase cycle is necessary for large cargo secretion, it is safe to conclude that Sar1 activation and inactivation must be tightly coordinated to form large COPII carriers.

## Evolutionary perspective on the ER exit site structure

Recent reports suggest that TANGO1 organizes ER exit sites in both *Drosophila* and mammalian cells (Liu et al., 2017; Maeda et al., 2017). Moreover, TANGO1 has been shown to be involved in general secretion other than collagen in *Drosophila* (Liu et al., 2017). However, in another report on *Drosophila* TANGO1 has been suggested to be only required for large cargo secretion and defects in small cargo transport with TANGO1 depletion were indirect due to ER stress caused by accumulated large cargoes (Rios-Barrera et al., 2017). Conversely, when both TANGO1 isoforms were depleted in mammalian cells, small cargo secretion was delayed but not blocked, probably because of the reduced number of functional ER exit sites. Moreover, TANGO1 directly interacts with Sec16 and functions as a scaffold to recruit Sec16 to ER exit sites (Maeda et al., 2017). The TANGO1 family proteins, TANGO1 and cTAGE5, have two isoforms each in mammalian cells. In contrast, *Drosophila* appears to possess only the long version of TANGO1 corresponding to TANGO1L. Thus, it is not easy to commensurate the phenotype observed in the two organisms. Further characterization is needed to uncover the functions of the TANGO1 family.

**Figure 2:**
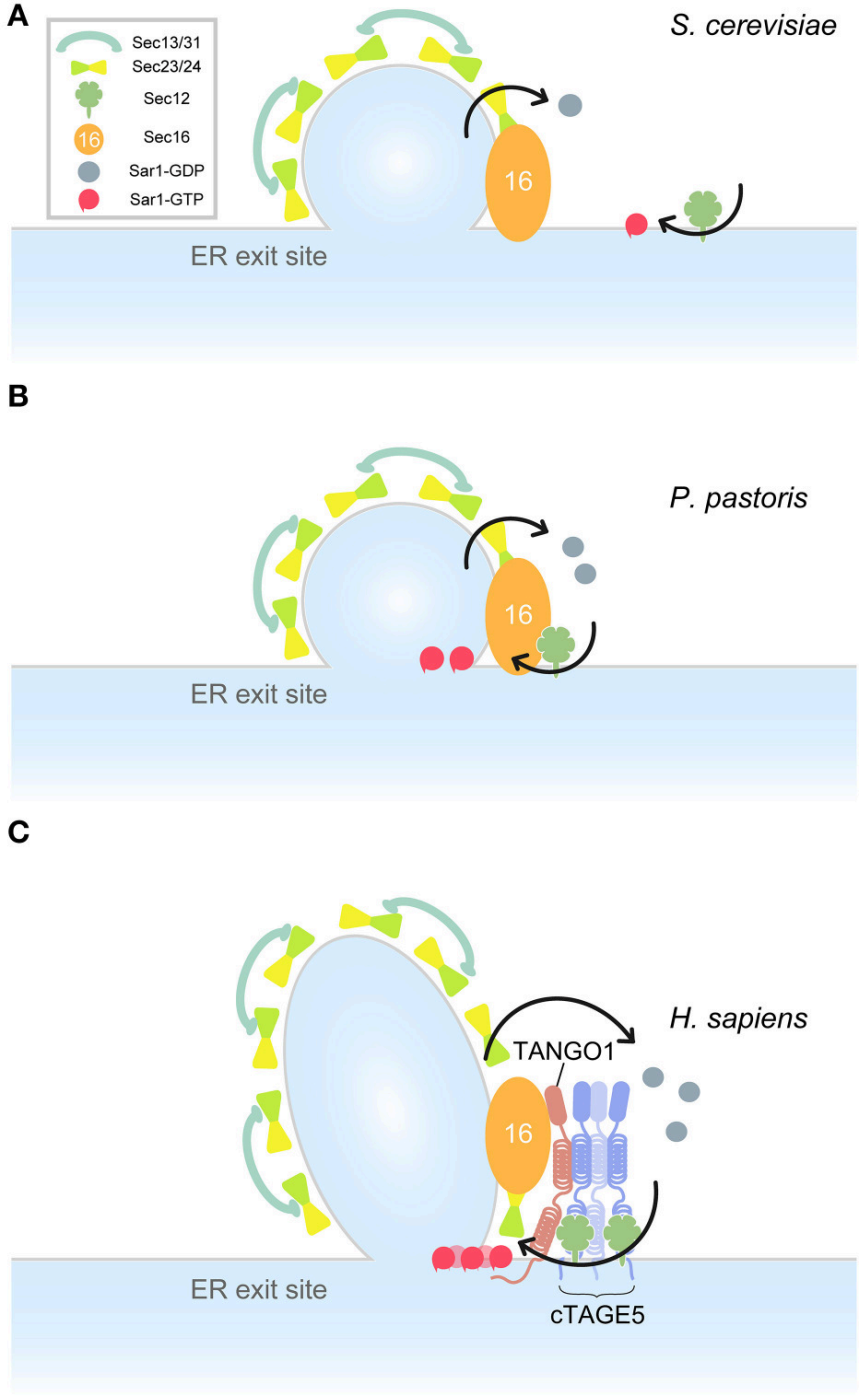
Model for ER exit site organization in different species. Arrows indicate Sar1 GTPase cycles. **(A)** A model for *S. cerevisiae* ER exit sites. Sec12 is dispersed throughout the ER. Sar1 accumulates at the rim of COPII-coated vesicles. **(B)** A model for *P. pastoris* ER exit sites. Sec16 is recruited to the ER exit site by interacting with COPII proteins. Sec12 is concentrated to the ER exit sites by interaction with Sec16. Sar1 accumulates at the ER exit sites. **(C)** A model for *Homo sapiens* ER exit sites. TANGO1 and Sec16 act as a scaffold to recruit COPII components and the membrane-spanning complex to the ER exit sites. Sar1 is efficiently activated by multiple Sec12 proteins within the cTAGE5/TANGO1/Sec12 complexes.

The core components of COPII proteins including Sar1, Sec12, Sec23/24, Sec13/31, and Sec16 are conserved from yeast to human. It is interesting to evaluate the ER exit site structure from an evolutionary perspective. In the budding yeast, *S. cerevisiae*, Sec16, Sec23/24, and Sec13/31 are localized at ER exit sites, although Sec12 shows a dispersed localization throughout the ER (Okamoto et al., 2012). Sar1 is modestly localized around the ER exit sites, but is excluded from other COPII proteins (Figure 2A) (Kurokawa et al., 2016). In a different budding yeast species, *P. pastoris*, Sec12 accumulates at ER exit sites by interacting with Sec16 (Montegna et al., 2012). In accordance with this, Sar1 localizes at the ER sites (Soderholm et al., 2004). Interaction with COPII proteins is required for Pichia Sec16 to localize correctly at the ER exit sites. Thus, Sec16 and COPII proteins are mutually required for organizing ER exit sites (Bharucha et al., 2013) (Figure 2B). In *Drosophila* and mammalian cells, however, Sec16 still localizes at the ER exit sites without COPII proteins, but depletion of Sec16 delocalizes COPII proteins from ER exit sites. These results suggest that there are other mechanisms of Sec16 recruitment to the ER exit sites in higher eukaryotes (Sprangers and Rabouille, 2015). A recent study has found that TANGO1 recruits Sec16 to the ER exit sites in mammalian cells (Maeda et al., 2017). Recruited Sec16 then accumulates COPII proteins at the ER exit sites. Moreover, TANGO1 forms a membrane-spanning megacomplex with multimeric cTAGE5 and Sec12 at the ER exit sites. Thus, TANGO1 acts as a scaffold to organize ER exit sites and recruits multiple Sec12 to ER exit sites for efficient activation of Sar1 GTPase (Maeda et al., 2017) (Figure 2C). The reported localization of TANGO1 around COPII coats as a ring-like structure supports this suggestion (Liu et al., 2017; Raote et al., 2017). It is interesting to speculate that *Drosophila* TANGO1 may also interact with Sec16. Because cTAGE5 is not present in this species, it would be worth testing whether *Drosophila* TANGO1 directly interacts with Sec12.

In summary, small GTPase Sar1 has a conserved role in COPII-coated carrier formation from yeast to human. Higher eukaryotes seem to introduce more elaborate mechanisms for the regulation of the Sar1 GTPase cycle, which facilitates secretion of diverse cargoes including large cargoes such as collagen.

## Author contributions

All authors listed, have made substantial, direct, and intellectual contribution to the work, and approved it for publication.

### Conflict of interest statement

The authors declare that the research was conducted in the absence of any commercial or financial relationships that could be construed as a potential conflict of interest.
